# Prevalence of Musculoskeletal Disorders and Self-Reported Pain in Artisanal Fishermen from a Traditional Community in Todos-os-Santos Bay, Bahia, Brazil

**DOI:** 10.3390/ijerph19020908

**Published:** 2022-01-14

**Authors:** Juliana dos Santos Müller, Eduardo Mendes da Silva, Rita Franco Rego

**Affiliations:** 1 Post-Graduate Program in Interactive Processes of Organs and Systems, Instituto de Ciências da Saúde, Federal University of Bahia, Salvador 40110-902, Brazil; 2 Department of Health and Services, Federal Institute of Santa Catarina, Florianópolis 88020-300, Brazil; 3 Instituto de Biologia, Federal University of Bahia, Salvador 40170-115, Brazil; dasilva@ufba.br; 4 Post-Graduate Program in Health, Environment and Work, School of Medicine, Federal University of Bahia, Salvador 40026-010, Brazil; 5 Instituto de Estudos em Saúde Coletiva, Federal University of Rio de Janeiro, Rio de Janeiro 21941-598, Brazil

**Keywords:** musculoskeletal disorders, musculoskeletal pain, shellfish gatherers, artisanal fishermen, small-scale fishery

## Abstract

Musculoskeletal disorders (MSDs) can be characterized from their occupational etiology and their occurrence; their chronicity generates negative repercussions for the health of workers, especially of artisanal fishing. To investigate the prevalence of generalized musculoskeletal disorders by body region and self-reported pain in a fishing population of northeastern Brazil, an epidemiological cross-sectional study was carried out in Santiago do Iguape, Bahia-Brazil, in 2017. The Brazilian version of the Nordic Musculoskeletal Questionnaire (NMQ), in addition to a questionnaire containing the socio-demographic and labor conditions were applied to a random stratified sample of 248 artisanal fisheries. There were 170 female shellfish gatherers and 78 fishermen, with a mean age of 36.7 years (SD = 10.5 years) and 43.3 years (SD = 11.8 years), respectively. The beginning of the labor activity was initiated at approximately 11 years of age. The average weekly income varied from 17.64 USD to 29.10 USD. The prevalence of MSD independent of occupation occurred in at least one body region in 93.5% and the presence of musculoskeletal pain/discomfort over the last seven days in 95.2% of the fishing workers. The highest prevalence of MSD was found in shellfish gatherers in: lower back (86.4%), wrist and hand (73.5%), and upper back (66.8%). In relation to the presence of pain in the last year, the frequency of pain was greater in the fishermen compared to the shellfish gatherers. The generalized severity of the MSD in 93.5% of this community of fishermen is evident, with emphasis in the following regions: lower back, wrist and hand and upper back in both groups, with occurrence of pain in more than one body region at the same time.

## 1. Introduction

Musculoskeletal disorders (MSD) have been recognized since the beginning of the 18th century as a series of maladies which have occupational etiological factors in common; however, only after the 1970s has MSD been screened through epidemiological methods and, since then, been present in the international scientific literature related to work and health problems [1]. From the occupational perspective, one of humanity’s oldest forms of work—that of artisanal fishing or small-scale fishing—has stood out in terms of exposure to the risks of MSD, especially because of the overburdening nature of manual labor [2], according to studies with a national focus [3,4]. 

In Brazil, these health problems were first described in 1973, and deemed to be the result of the excessive use of the musculoskeletal system, combined with the lack of time for recuperation, suffered by individuals under certain conditions of work [5]. MSDs, also known as cumulative traumatic disorders, are pathologies which afflict a diverse group of professions [6]. According to the Brazilian Ministry of Health, the following categories are among those most at risk for MSD, cleaning staff, operators of fixed machines, production line suppliers, cooks in general, vehicle assemblers (assembly line), domestic workers, bricklayers, check out cashiers, welders, and lorry drivers [7]. However, this data does not represent the contingent of small-scale fishing workers, since it only considers formal Brazilian workers, which highlights the invisibility of this group in the official national statistics. 

The work processes of fishermen in the Todos-os-Santos Bay (BTS) have been in place for generations with few modifications up until today, which are inherent to the socio-cultural and environmental conditions, with the only difference being the contemporary capitalist system of production through which the fishermen depend on middlemen [8]. Fishing production is individual, low-skilled and is performed with rudimentary tools, and it is through this production that the fishermen survive from the sale of the product of their labor to an intermediary, who purchases the catch and resells it to the retailer. In terms of the gender division of work, men predominantly work with fish and shrimp and other marine animals, while women work principally in the collection, transport and preparation of the shellfish, as well as assuming responsibility for habitual domestic tasks [8].

Subsistence fishing is an important economic activity, especially in rural areas, but on a global scale, the number of subsistence fishermen is little quantified nor is the importance of this activity for these families recognized [9]. According to specific legislation, artisanal fishing is that which is practiced directly by the professional fishermen, autonomously or in a family economic regime, with their own means of production or through a partnership contract, on land or in a small sized vessel [10] and encompasses all the processes of fishing, exploitation, cultivation, conservation, processing, transport and commercialization of fishing resources.

Given the coastal geographical characteristics in the BTS, fishing has an enormous social, local and regional importance and has traditionally been an important source of subsistence for many populations, the majority of whom depend on fishing and activities related to it. Therefore, in Brazil, artisanal fishing, or small-scale fishing, is an important form of work which occupies the fourth position in terms of fishing production in the region of Latin America and the Caribbean [11] and artisanal fishing is responsible for 45% of national production with a contingent of workers of one million fishermen, 99% of whom are categorized as artisanal fishermen recorded in Brazil up until the year 2011 [12]. The State of Bahia has an expressive number of fishermen registered in the latest published official data and reaches more than 100,000 workers [13].

In spite of the importance of recognizing the health of these workers, there is a lack of understanding about the extent of problems related to it [14] and where muscular overburdening occurs in the areas of the neck, shoulder, back, upper limbs, lower back and wrists [5]. 

According to Remmen and contributors (2020) [15], in the systematic review of MSD in professional fishermen, there was a general MSD prevalence ranging from 15% to 93%. Other international studies have also demonstrated the presence of symptoms of MSD and musculoskeletal pain in fishermen from those countries: United States [16], Turkey [17], India [18], Norway [19], Denmark [20], Greece [21], Finland [22], China [23], and Spain [24], which highlights the difference in fishing activity in those countries and the general fishing industry, in relation to the predominant Brazilian scenario of small-scale artisanal fishing.

In the systematic review of fishermen in Latin America, in 2019 [25], the authors concluded the scarce number of epidemiological studies referring to occupational injuries, and the need to establish preventive policies on fishing activities; in this scenario, Brazilian studies accounted for about 55% of carried out studies in Latin America and were included in this systematic review, but far from ideal for the complex debate on health problems and their repercussions on the health of fishery workers.

Initially, the definition of health risk parameters for fishing workers in Bahia and consequently the occurrence of MSD was carried out based on qualitative ethnographic and ergonomic methodologies, with subsequent clinical diagnosis to understand the work and possible health risks of shellfish gatherers in Bahia de Todos-os-Santos, which culminated in epidemiological studies of a quantitative nature [3]. In these analyses, according to Pena and Gomez (2014) [26], the ergonomic evidence of MSD risks in artisanal fishers is mainly related to the overload of tasks with excessive movement and repetitive efforts, imposed by accelerated rhythms and dramatic social conditions for survival.

In Brazil, the diagnosis of DME [5] is established from a multidisciplinary consultation with the integration of epidemiological data to promote a diagnostic hypothesis. The main related factors are the biomechanical, cognitive, sensory, affective and work organization aspects. The classic literature points out the following risk factors associated with the causality of MSD: physical demands such as repetitiveness, inadequate postures and strength [1,27], but in the study by Costa and Vieira (2010) [27,28], the main evidence about risk factors were: heavy physical work activities, smoking, high body mass index, high psychosocial work demands, and the presence of comorbidities, including excessive repetition, uncomfortable postures, and heavy lifting (load).

The work process of artisanal fishermen, considering men and women, presents similarities in the raw material (fish and shellfish available in nature), in the environment and in the work organization (rudimentary technical process; family work; there is no salary; and use of traditional knowledge) and in relation to the final product manufactured, fish and shellfish, ready for sale and consumption [8].

In this way, epidemiological studies with these workers are fundamentally important in order to describe the health problems in this segment of informal workers. Therefore, this study aims to verify the prevalence of generalized musculoskeletal disorders per body region and self-reported pain, characterized according to the occupation of a fishing population in the northeast region of Brazil. 

## 2. Materials and Methods

**Figure 1:**
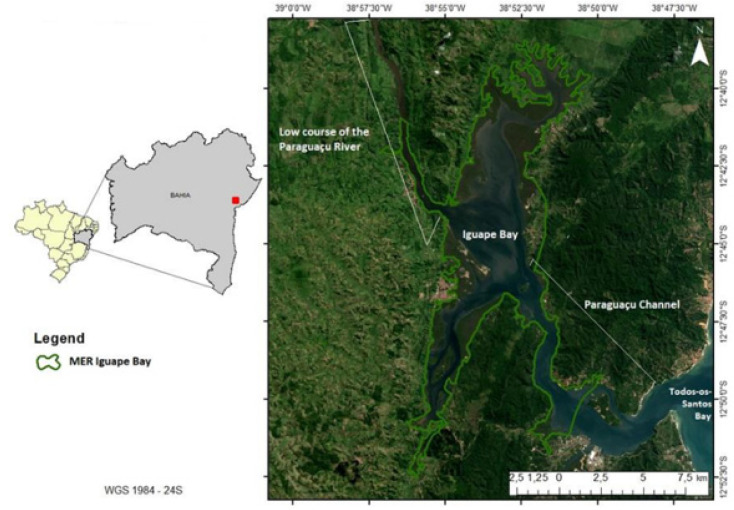
Location map of the Paraguaçu River estuary and area limits of Marine Extractive Reserve Iguape Bay. Author: Veloso; Moraes [32].

A cross-sectional study was carried out to survey the prevalence of generalized musculoskeletal disorders per body region and self-reported pain with artisanal fishermen (here including female shellfish gatherers and fishermen); residents of the district of Santiago do Iguape, which belongs to the municipality of Cachoeira in the State of Bahia (Brazil) (MAP, Figure 1). The investigation of these informal workers in that location emerged from the principle of community-based participatory research, this method enables all partners to contribute with their knowledge, sharing the responsibility and ownership; improves understanding of a given phenomenon; and it integrates the knowledge gained with actions to improve the health and well-being of community members, such as through actions, interventions and policy changes. The demand for this research came from the fishing community to the University [29].

**Figure 2:**
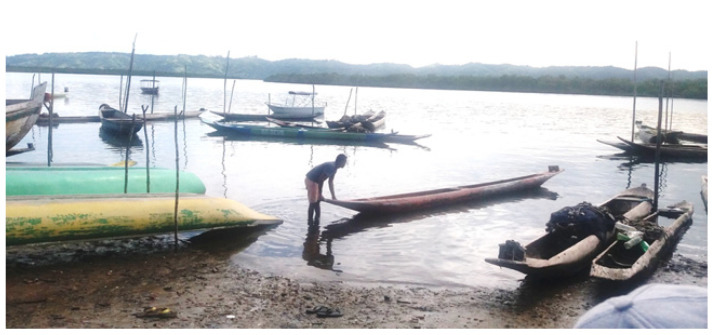
Fishing activities in small boats in Santiago do Iguape, Bahia, Brazil. Author: Research data.

The district referred to is part of a federal Marine Extractive Reserve of the Iguape Bay (MER) with an area of 24,892.854 acres, and is part of a larger estuarine complex, the Todos-os-Santos Bay. If this is federal the legal principle has been already indicated, which establishes the guarantee for self-sustainable exploitation, as well as the conservation of renewable natural resources on the part of the resident traditional communities [30]. The fishing community has around 2500 inhabitants, and fishing is the main source of sustenance for the population (Figure 2) [31].

### 2.1. Sampling and Criteria Used

In order to characterize the fishing population, official data recorded in the Maritime Extractive Reserve of the Bay of Iguape from 2017 was used. Afterwards, a drawing of artisanal fishermen was carried out, using the table of random numbers for a probabilistic sampling approach, stratified by sex and without replacement. For the sample calculation, the prevalence of musculoskeletal disorders of 50% was adopted according to previous studies involving artisanal fishermen [33,34,35], with an alpha error of 5% in a total population (N) of 537, calculating a margin of loss or refusal of 10%, resulting in a final sample of 248 artisanal fishermen. 

The inclusion criteria previously established were: being 18 years of age or older and having been engaged in the activity (fishing) for at least one year. The drawn workers who were not engaged in artisanal fishing activities had the opportunity to participate. If they had diseases that have a possible relationship with Musculoskeletal Disorder, then, in order to minimize the bias of healthy fishermen they were justifiably added. In particular, male fishermen were more likely to have related diseases. Participants gave consent to participating in the study by signing the Informed Consent Form. Selected workers were invited by phone, letter or in person.

This study assumes the historical recognition of the centrality of women in the activity of shellfish gathering and the men in maritime fishing, in small vessels, as previous studies performed in Todos-os-Santos Bay have shown [2,36], and so in this study, we refer to the women as shellfish gatherers and the men as fishermen. 

It should be emphasized that the proposal for this investigation was approved by the Committee on Ethics in Research of the Medical School of Bahia at the Federal University of Bahia (3066.8570).

### 2.2. Data Collection Instruments

Data collection occurred in the months of May and June 2017, followed by the training of the research team, as well as a previous pilot study, which were undertaken. The collected data entailed structured questionnaires/interviews elaborated by the researchers along the following lines: identification of the fishermen; socio-demographic information; work history; self-reported information about present health conditions. 

To evaluate the symptomatology of MSD, a broadened, translated and validated Brazilian version of the Nordic Questionnaire Musculoskeletal NQM [37]. This questionnaire was developed in order to standardize the measurement for reports of musculoskeletal symptoms, and therefore facilitate the comparison of results between studies. The authors of the questionnaire do not recommend it as the basis for clinical diagnosis, but rather for identification [27,38]. In this article, all the areas of the body were used in a general and amplified manner classified in 12 anatomical regions. It should be emphasized that the NQM has shown high reliability in previous studies [27,37].

To characterize the presence of self-reported presence of pain and musculoskeletal discomfort, a positive report over the last 12 months in at least one of the 12 anatomical areas investigated, was established as a benchmark. 

### 2.3. Analysis Plan

To define cases of MSD, the reporting of pain or discomfort over the last 12 months was used, that lasted for more than a week or minimal monthly frequency, not caused by a serious injury and showing a gravity of ≥3, on an ordinal scale of 0 to 5, with explanatory verbal qualifiers in the extremities, 0 = no pain, 5 = unbearable pain, or, which determined the seeking of medical attention or official or not absence from work or changing of work, in at least one of the following regions: neck, shoulder, elbow, forearm, wrist/hand, upper back, lower back, thigh, knee, leg, ankle and foot [27]. 

To characterize the pain or discomfort, the first question in the Nordic Questionnaire was about symptomatology over the last 12 months. Generalized MSD was characterized as the presence of cases in which the number of regions affected was greater or equal to 1 (one), arbitrarily to the criteria of the researchers involved and similar to the criteria used in other studies [39]. 

### 2.4. Statistical Analysis

The dispersion measures and central tendency for the continuous variables and measures of frequency for the categories were calculated. To calculate the Prevalence Ratio (PR), it was arbitrarily assumed as a reference of the occupation the category of fishermen (male gender). The prevalence of MSD and generalized self-reported pain were calculated according to the occupations with the respective global confidence intervals of 95%, adjusted for the two simultaneous null hypotheses. The same method was used to calculate the confidence intervals for the prevalence ratios for MSD and self-reported pain according to occupation. 

The prevalence of MSD and self-reported pain by body region was calculated, according to the occupations and with the respective global confidence intervals of 95%, adjusted for 12 simultaneous null hypotheses. All the adjusted confidence intervals cited were calculated using the Šidák method [40]. 

The Šidák correction (**α**global/Number of H0)1/2) is slightly less restrictive than the Bonferroni correction (**α**global/Number of H0), thus slightly increasing the power of the test. This adjustment was necessary due to the probability of incorrectly rejecting (false positive) a null hypothesis when simultaneously testing null hypotheses [41]. 

For the number of body regions afflicted by MSD according to occupation, the prevalence and respective global confidence intervals of 95%, adjusted by the Sison and Glaz method for multinomial proportions, were computed, given the multinomial nature of the distribution of probability of MSD for number of regions [42]. The analyses were performed in the R statistical environment version 3.6.3 [43]. 

## 3. Results

The sample of artisanal fishermen of Santiago do Iguape, investigated in this study, was made up of 248 individuals, of whom 170 were female shellfish gatherers (FSG) and 78 were fishermen (FS). In terms of the socio-demographic characteristics presented in Table 1, of note is the average age of 36.7 (SD = 10.5) for the female shellfish gatherers, and an average age of 43.3 (SD = 11.8) for the fishermen. The average hours worked per day was 8.8 h (SD = 1.9) and 9.1 (SD = 3.0) for the two groups, respectively. In both occupations, work was initiated at the age of around 11. The average weekly income varied from 17.64 USD to 29.10 USD. It should be emphasized that the great majority of the two groups was self-declared skin color Black and Brown (96.4% and 94.9%) and a marital status categorized as single/separated or widowed. In terms of schooling, in both the groups, about 30% had incomplete middle school. 

### 3.1. General Health Conditions of the Artisanal Fishermen

Regarding the prevalence of musculoskeletal disorders independent of occupation, there was evidence of the occurrence of this in at least one region of the body in 232 individuals, which corresponded to 93.5% [89.2–96.6]%, as well as pain/discomfort experienced over the last seven days in 236 individuals with a prevalence of 95.2% [91.2–97.7]%. In both the characteristics that appeared, when considering occupations, as in Table 2, health problems were elevated, without, however, differences between the prevalence of MSD and pain: still, the occurrence of a percentage of higher than 90% of those investigated in this study should be emphasized.

### 3.2. Prevalence of Musculoskeletal Disorders (MSD) by Body Region

The descriptive results show that there was an important difference between the prevalence of MSD per body region in all regions of the body, always higher in the shellfish gatherers, except in the elbow. However, due to the size of the sample, the confidence intervals were amplified. 

**Figure 3:**
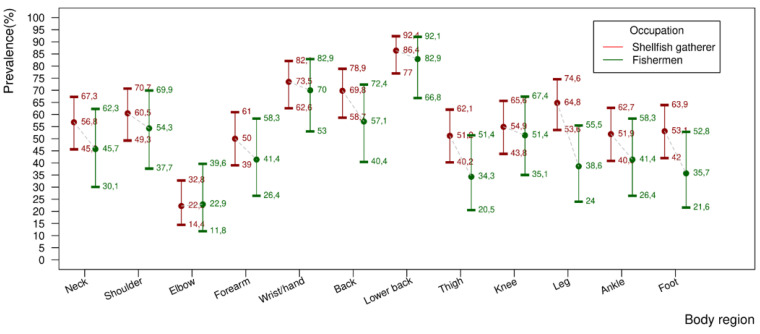
Prevalence and confidence intervals of Musculoskeletal Disorders (MSD) by body region in artisanal fishermen in the municipality of Cachoeira, district of Santiago do Iguape, Bahia, Brazil. L. adjusted for 12 simultaneous tests using the Šidák method.

Considering the prevalence of MSD per body region shown in Figure 3 for both the groups analyzed in an isolated manner and according to the findings, the highest prevalence of MSD in the shellfish gatherers was described in the following regions: lower back (86.4%); wrist and hand (73.5%); upper back (66.8%); in the male fishermen group, the same regions were affected: lower back (82.9%); wrist and hand (70.0%); and upper part back (57.1%). 

### 3.3. Prevalence of MSD in Multiple Regions of the Body in Artisanal Fishermen

According to Table 3, the simultaneous occurrence of MSD in more than one region of the anatomy, independent of occupation, varied between 8.5% and 10.7%. It is well-known that the prevalence of MSD, according to the number of regions, does not follow a mathematical pattern of growth according to the increase in the number of regions involved.

### 3.4. Presence of Symptoms of Pain and Discomfort in the Last 12 Months by Body Segment

**Figure 4:**
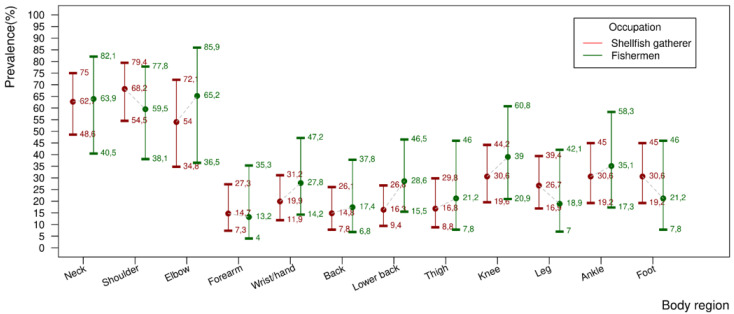
Presence of Symptoms (pain and discomfort) over the last 12 months by body segment in artisanal fishermen in the municipality of Cachoeira, district of de Santiago do Iguape Bahia, Brasil. L. adjusted for 12 simultaneous tests using the Šidák method.

Regarding the presence of pain in the last year, according to Figure 4, of note is that fishermen and shellfish gatherers experienced pain in the last year in all the body regions, and the frequency of pain was greater among fishermen than shellfish gatherers in the majority of the areas studied. 

For the classification of MSD, a positive response for pain is vital (dependent) and, thus, the areas most affected by pain were the area around the neck, elbow and back for both the groups. Even though this was not part of the objective of the study, an association between MSD and self-reported pain was observed of 0.86 * [0.69–1.00] 95% and for fishermen of 0.85 * [0.60–1.00] 95%, showing a strong association between these problems. 

## 4. Discussion

This study aimed to verify the prevalence of generalized musculoskeletal disorders per body segment, as well as musculoskeletal pain within the occupation of artisanal fishing, through the application of quantitative, epidemiological instruments. This methodological approach according to occupation, proposed within the scope of a contingent of fishing workers in a city in the northeast of Brazil, was used due to what had been a gap in scientific production. The findings show the difference in self-reported musculoskeletal pain in female shellfish gatherers and fishermen and also reaffirm the similarity of the exacerbated occurrence of generalized MSD in this artisanal fishing community. 

The paucity of information about the presence of health problems, especially MSD and musculoskeletal pain in artisanal fishermen, can be understood, in part, by the manner in which this activity is performed in Brazil, which is widespread and complex, with social, political, institutional, economic and environmental specificities intrinsic to each place [12], as well as epidemiological invisibility due not only to under-reporting, but also the absence of adequate reporting in the informal labor sector [26]. This problem objectively shows the reality of artisanal fishermen in the Brazilian northeast, who perform their activity in limited areas, using rudimentary instruments, outside the formal economy and with few resources available for their own subsistence or for taking care of maintaining health care. 

Within the findings, in the socio-demographic criteria, one can see that the monthly income of fishing workers is 70 USD for the female shellfish gatherers and 117 USD for fishermen, which corresponds to 23.43% and 40.38% of the minimum salary in the country in 2017, respectively, as well as low levels of schooling, and early start to working in childhood (average of 11.5 years-old). According to Pena and collaborators (2011) [4], these variables generate social deprivation, since it imposes an intense rhythm of work to produce more products to sell and consequently greater damage to health, according to the ethnographic study. 

It was possible to verify that low levels of schooling can be responsible for the ineffectiveness of public policies for fishing, as well as the fact of how easy it is for people, with absolutely no options, to start performing this fishing activity, stoking the paradigm of fishing and poverty [44]. The vulnerability and worsening of health conditions is increased due to the invisibility of this population to society and to the health systems. In addition, within the context of this study, a community-based participative research, one questions the complex idea of equity in health for fishing artisanal professionals. 

The findings described before are critical and show the vulnerability and the difficulty in maintaining health in this traditional population and for the symptomatologic analysis of generalized MSD and by body region. Both groups, shellfish gatherers and fishermen, were afflicted in all anatomical regions, with extreme prevalence, with a minimum MSD threshold of 22.2% and a maximum of 86.4%. Within the findings, the most expressive prevalence of MSD in shellfish gathers/fishermen was in the: lower back region (86.4%/82.9%), followed by wrist and hand (73.5%/70.0%) and the upper back (69.6%/57.1%). The similarity to another study performed with oyster fishermen in Taiwan should be emphasized [23], where it was found that the oyster fishermen also had MSD in their backs, hands, wrists, shoulders and elbows. 

Descriptively, there is a higher prevalence of MSD in shellfish gatherers, very similar to previous studies performed in the Brazilian northeast, in which the authors also found a high prevalence of generalized MSD [35] among shellfish gatherers, as well as pain in the upper limbs [45], lower back region [45], all related to the practice of fishing [34]. Evidence of MSD symptoms were also found in studies with fishermen, for example in the United States (42.8–83.3%) [16], Turkey (84%) [17], Norway (33%) [19] and Spain (65.5–88%) [24].

Within this context, two aspects are shown: (1) artisanal fishermen have a high prevalence of MSD (generalized/body region) and pain in both the activities; (2) the symptomatology dependence of musculoskeletal pain, even when strongly associated, were distinct in some body segments per occupation. Therefore, this clearly suggests that the activity of fishing is physically damaging and can cause or aggravate musculoskeletal disorders, similar to the findings among Danish fishermen [20], and that the work process involved in fishing spurs the worsening of MSD. 

Elevated prevalence of MSD was identified in both the occupations. Among the female shellfish gatherers, the frequency was of 86.4% and among fishermen, 82.9%, results similar to the study undertaken by Couto and collaborators [34] (2019), with shellfish gatherers in northeastern Brazil. In the study of Fragoso and collaborators [46] (2018), performed with fishermen in the Brazilian Amazon region, 50% levels of MSD were found in the lower back region, showing the same severe occurrence of that in fishermen in Santiago do Iguape, where there is a prevalence of 82.9%; still, it should be emphasized that these findings were higher than, for example, those of the study undertaken with fishermen in Nigeria [45], where the MSD in the lower back was of 68.23%. 

One hypothesis for these alarming findings is the overwhelming presence of ergometric risks for these artisanal fishing workers, such as: physical force, repetitive/monotonous movements and the absence of breaks. The analysis of factors associated with MSD can justify the exacerbated frequencies found, both for MSD and pain, as well as the anatomical predilections among the groups in the descriptive analysis. For example, according to the Regulation Norm (NR) 17 [47], which regulates Brazilian labor law, a pause is necessary during a work shift to recuperate static and dynamic muscular overburdening. The inexistence or limited nature of work pauses is evident for the informal worker, especially those who depend on natural resources for their subsistence [34,45,48].

With the use of the Nordic Questionnaire with systematic revision [49], from 2017, only two studies were carried out describing fishing professionals, showing the lack of visibility of scientific studies which involve female artisanal fishing professionals and factors associated with them [50]; further, the fact that the activity of fishing is physically demanding and affects almost all the musculoskeletal system was ratified. The factors impact the gravity of the findings in one of the articles consulted [48], as well as showing how few epidemiological-based studies there are in Brazil which portray this professional category and the relations between health and artisanal labor processes [48].

Another important point is the study performed with Norwegian fishermen [19] who demonstrated MSD, in elevated rates compared to the general Norwegian population, highlighting the negative health effects when executing this kind of work activity. Parallel to Brazil, if we compare the prevalence of MSD in artisanal fishermen in Todos-os-Santos Bay with other fishing professionals (industrial), the prevalence of MSD and musculoskeletal pain are exacerbated in all bodily regions, such as upper limbs and neck, for the Norwegians, 33% of the studied sample complained of pain, whereas the rate for Brazilian artisanal fishermen is around 70%. 

The difference found in terms of the self-reported complaints of pain in relation to the presence of MSD in this population shows a low concern for the presence of pain, perhaps because it is a chronic condition or also apparently normal, or in other words, an adaptation related to work. The findings show that the regions with higher prevalence of pain were: the neck, elbow and back, which diverge from the cases of MSD analyzed. The results can suggest that for this professional group, a deeper analysis beyond the current biomedical model [51] is necessary, since factors that cause chronic pain impede the continuance of their autonomy, something indispensable for artisanal fishing. 

Still from this perspective, the perception of pain over the last seven days was described more by the fishermen than the female shellfish gatherers. According to Almeida and collaborators [52], woman have a greater predisposition to attribute body sensations to physical illnesses, however, due to the multi-causality, the biological, bio-mechanical behavioral factors will be influential in determining them [53]. For Ramiser-Maestre [54], it is necessary to evaluate the perception of pain through a biopsychological model, especially when considering analysis by gender; in this way, the study cited [52] suggests that women have high pain perception, but better adapt to chronic pain, and as a consequence, have better functional capacity in relation to daily activities. In the findings of this study, it can be observed that 92.5% of artisanal fishermen reported musculoskeletal pain in at least one region of their bodies over the last year, the most afflicted parts being the regions of the wrist/hand, upper and lower back, which is closer to the findings of other scientific investigations [20,21,22,23,24,25,26,27,28,29,30,31,32,33,34,35,36,37,38,39,40,41,42,43,44,45,46,47].

Within the panorama presented, in a quantitative manner, the high prevalence of MSD and musculoskeletal pain in artisanal fishermen is evident throughout the body of those groups analyzed, which represents an important socio-economic problem, since its occurrence is accentuated and requires the maintenance of health in order to execute their work to ensure the subsistence of their families. 

Thus, the need to give visibility to this health problem is evident, through its recognition and its effective official notification, as well as having strategies for analyzing and managing the chronicity and functional incapacity that may occur in this group of workers. In addition, the health inequities, directly expressed by socioeconomic inequalities, and the occupation of these workers, reinforce the demand for more research that contributes to the advancement of knowledge about this complex network of causal relationships, which are manifested in the dimension of the health-disease process, and in this occupational hazard, particularly in these communities that traditionally have artisanal fishing and related activities as their source of subsistence, but which, surprisingly, produce a volume of fish that quantitatively places Brazil in a prominent position in Latin America and the Caribbean. 

### 4.1. Strengths and Limitations 

The limitations of the current study are concentrated firstly in the input of the methodological design applied through reverse causality, the fragility of time and the doses of exposition to factors which possibly result from MSD, as well as the effect of the researcher, visible in any community-based participative research undertaken with this population group. 

### 4.2. Recommendations

The paucity of studies about Brazilian artisanal fishermen and their peculiarities shows the necessity of developing prospective epidemiological studies, as well as demonstrating the necessity of having official recognition of the problems of the Brazilian fishing population, since there is a gap in data and scientific evidence with comparative scenarios between men and women. 

## 5. Conclusions

The current study demonstrated and reinforced the gravity of the generalized MSD in 93.5% of the fishing community, particularly in these regions: lower back, wrist and hand and the upper back in both groups, further reiterating that it usually occurs in more than one region of the body at the same time. Musculoskeletal pain is strongly associated to the presence of MSD in all body regions, although it is more frequent in: the neck, elbow and shoulder. Musculoskeletal pain is more prevalent in fishermen when compared with female shellfish gatherers.

Thus, the necessity of greater visibility for this health problem is evident, from official notification to strategies of analysis and management of chronic pain, and functional incapacity that can occur with this group of workers, who depend on maintaining their health to generate and guarantee their subsistence.

## Figures and Tables

**Table 1:** Socio-demographic characteristics and health conditions in artisanal fishermen in Santiago do Iguape, Bahia, Brazil (*n* = 248).

Socio-Demographic Characteristics	Shellfish Gatherers	Fishermen
	Average (SD)	Average (SD)
Age in years	36.7 (10.5)	43.3 (11.8)
Weekly income in reais (R$)/USD *	57.0 (35.7)/17.64	94.6 (55.9)/29.10
Daily number of hours worked shellfish gathering/fishing	8.8 (1.9)	9.1 (3.0)
Days worked in a week	4.7 (1.4)	5.3 (1.5)
Age when starting shellfish gathering/fishing	11.7 (3.7)	11.1 (3.2)
Sex	N (%)	N (%)
Female/Male	170 (100%)	78 (100%)
Place of birth		
Santiago do Iguape	71 (41.7)	44 (56.4)
Santo Amaro	11 (6.4)	6 (7.6)
Salvador	12 (7.0)	7 (8.9)
Cachoeira	65 (38.2)	19 (24.3)
Other locations	11 (6.4)	2 (2.5)
Self-declared skin color	N (%)	N (%)
Yellow	1 (0.6)	1 (1.3)
White	2 (1.2)	2 (2.6)
Indian	1 (0.6)	3 (3.8)
Black	135 (79.4)	52 (66.7)
Brown	29 (17.0)	22 (28.2)
Marital status	N (%)	N (%)
Married (a)/Dating (a)/Live together (a)	71 (41.8)	31 (39.70)
Single (a)/Separated (a)/Widow (a)	99 (58.2)	47 (60.3)
Schooling	N (%)	N (%)
No schooling	5 (2.9)	8 (10.3)
Primary school	27 (15.9)	15 (19.2)
Incomplete middle school	49(28.8)	24 (30.8)
Complete middle school	9 (5.3)	5 (6.4)
Complete high school	56 (32.9)	14 (17.9)
Incomplete high school	24 (14.1)	11 (14.1)
Incomplete undergraduate studies	1 (1.3)	0 (0.0)

Source: Research data. * Approximately in dollars in the year 2017.

**Table 2:** Prevalence and prevalence ratio of musculoskeletal problems in artisanal fishermen from Santiago do Iguape, Bahia, Brazil (*n* = 248).

Musculoskeletal Problems	Shellfish Gatherer	Fisherman	
	*n* (%)	*n* (%)	Prevalence Ratio PR and Confidence Interval [CI] ^2^
MSD in at least one body region ^1^	162 (95.3)	70 (89.7)	1.06 [1.00–1.13]
Musculoskeletal pain or discomfort experienced in the last 7 days in at least one body region ^1^	164 (96.5)	72 (92.3)	1.05 [0.99–1.10]

Source: Research data. Key: ^1^ CI at global 95% adjusted to the simultaneous test of two null hypotheses using the Šidák method ^2^.

**Table 3:** Prevalence of MSD in multiple regions of the body in artisanal fishermen in the municipality of Cachoeira, district of de Santiago do Iguape Bahia, Brazil.

Number of Regions	Shellfish Gatherer (170)		Fisherman (78)			TOTAL	
	*n*	Prevalence (%)	CI *	N	Prevalence (%)	CI *	*n*
0	0	0.0	[0.0–6.5]	0	0.0	[0.0–9.9]	0.0
1	16	9.4	[4.1–15.9]	7	9.0	[1.3–18.9]	21
2	9	5.3	[0.0–11.8]	11	14.1	[6.4–24.0]	20
3	14	8.2	[2.9–14.7]	5	6.4	[0.0–16.3]	19
4	7	4.1	[0.0–10.6]	7	9.0	[1.3–18.9]	15
5	12	7.1	[1.8–13.5]	5	6.4	[0.0–16.3]	17
6	10	5.9	[0.0–12.3]	8	10.3	[2.6–20.1]	17
7	12	7.1	[1.8–13.5]	3	3.8	[0.0–13.7]	15
8	19	11.2	[5.9–17.6]	6	7.7	[0.0–17.6]	25
9	12	7.1	[1.8–13.5]	6	7.7	[0.0–17.6]	18
10	18	10.6	[5.3–17.1]	2	2.6	[0.0–12.5]	20
11	18	10.6	[5.3–17.1]	5	6.4	[0.0–16.3]	23
12	15	8.8	[3.5–15.3]	5	6.4	[0.0–16.3]	20

L Source: Research data. L Key: * In this study, at least one affected region was considered. * Confidence interval of 95% adjusted for 12 simultaneous tests by the Sison and Glaz method.

## Data Availability

The data presented in this study are available on request from the corresponding author.
